# Infliximab reduces Zaprinast-induced retinal degeneration in cultures of porcine retina

**DOI:** 10.1186/s12974-014-0172-9

**Published:** 2014-10-10

**Authors:** Cristina Martínez-Fernández de la Cámara, Lorena Olivares-González, David Hervás, David Salom, José M Millán, Regina Rodrigo

**Affiliations:** Sensorineural Disorders, Health Research Institute-La Fe, Valencia, Spain; Biostatistics Unit, Health Research Institute-La Fe, Valencia, Spain; Department of Ophthalmology, La Fe University Hospital, Valencia, Spain; Centre for Biomedical Network Research on Rare Diseases (CIBERER), Madrid, Spain; Genetics Unit, La Fe University Hospital, Valencia, Spain; Laboratory of Molecular, Cellular and Genomic Biomedicine, Institute of Health Research- La Fe, Avenida Fernando Abril Martorell 106, 46026 Valencia, Spain

**Keywords:** Retinal degeneration, Inflammation, Infliximab, Oxidative stress, TNFα, Poly(ADP-ribose), *caspase-3*, Retinitis pigmentosa, Photoreceptor death

## Abstract

**Background:**

cGMP-degrading phosphodiesterase 6 (PDE6) mutations cause around 4 to 5% of retinitis pigmentosa (RP), a rare form of retinal dystrophy. Growing evidence suggests that inflammation is involved in the progression of RP. The aims of this study were to corroborate the presence of high TNFα concentration in the eyes of RP patients and to evaluate whether the blockade of TNFα with Infliximab, a monoclonal anti-TNFα antibody, prevented retinal degeneration induced by PDE6 inhibition in cultures of porcine retina.

**Methods:**

Aqueous humor from 30 patients with RP and 13 healthy controls were used to quantify the inflammatory mediators IL-6, TNFα, IL-1β, IL-10 by a multiplex enzyme-linked immunosorbent assay (ELISA) system. Retinal explants from pig were exposed to Zaprinast, a PDE6 inhibitor, for 24 hours in the absence or the presence of Infliximab. Cell death was evaluated by TUNEL assay. The number and distribution of *caspase-3* positive cells, indirect poly(ADP)ribose polymerase (PARP) activation and glial fibrillary acidic protein (GFAP) content were visualized by immunolabeling. Antioxidant total capacity, nitrites and thiobarbituric acid reactive substances (TBARS) formation were determined to evaluate antioxidant-oxidant status.

**Results:**

IL-6 and TNFα concentrations were higher in the aqueous humor of RP patients than in controls. Infliximab prevented retinal degeneration, as judging by the reduced presence of TUNEL-positive cells, the reduction of *caspase-3* activation and also reduction of glial activation, in an *ex vivo* model of porcine retina. Additionally, Infliximab partially reduced oxidative stress in retinal explants exposed to Zaprinast.

**Conclusions:**

Inflammatory mediators IL-6 and TNFα were elevated in the aqueous humor of RP patients corroborating previous studies suggesting sustained chronic inflammation. Our study suggests that TNFα is playing an important role in cell death in an *ex vivo* model of retinal degeneration by activating different cell pathways at different cell layers of the retina that should be further studied.

**Electronic supplementary material:**

The online version of this article (doi:10.1186/s12974-014-0172-9) contains supplementary material, which is available to authorized users.

## Background

Retinitis pigmentosa (RP) is a common form of rod-cone dystrophy, constituting the largest Mendelian genetic cause of blindness in the developed world. Patients with RP typically loose night vision in adolescence, peripheral vision in young adulthood, and central vision later in life due to progressive loss of rod and cone photoreceptor cells. Photoreceptor cell death starts with rod photoreceptor degeneration and eventually cone cell death that is the major problem affecting RP patients, because it leads to loss of central vision [1]. More than 60 genes, including phosphodiesterase 6 (*PDE6*) subunit genes, have been identified to date that, when mutated, cause different forms of non-syndromic RP [2-7].

Although RP is a genetic disease, increasing evidence in patients and animal models suggests that oxidative stress and inflammation, especially TNFα, contribute to its pathogenesis, independently of the genes mutated [8-10]. Some reports show the presence of sustained chronic inflammatory reaction including elevated TNFα levels in the eyes of RP patients [11] and *rd10* mice [12]. TNFα is a pleiotropic cytokine essential for the induction and maintenance of the inflammatory immune responses [13] that is also upregulated in inflammatory ocular diseases, including Adamantiades-Behcet disease [14], retinal vascular tumors [15], neovascular age-related macular degeneration [16], uveitis [17], glaucoma [18] and ischemic retinopathy [19].

TNFα mediates a broad range of cellular activities, including proliferation, survival, differentiation, inflammation and cell death. In the retina, TNFα is likely to be secreted from activated macrophages, astrocytes, microglial cells and retinal Müller glial cells. TNFα can trigger several well-characterized death-promoting (caspase-dependent and caspase-independent cell death) and survival-promoting pathways, depending upon the predominating signaling pathway in the particular cell type [20]. TNFα binding to cell surface receptors such as TNFR1 mediates activation of initiator caspases (*caspase*-*8*, *caspase-10*) and finally triggers cleavage of effector caspases (extrinsic pathway of cell death) [21]. TNFα is also involved in the intrinsic pathway of cell death that is initiated by cellular and DNA damage which particularly involves mitochondria. Finally, TNFα can also activate a subset of programmed necrosis called necroptosis. The mechanism that leads cells to undergo apoptosis or necroptosis and the mechanism that mediates the execution of necroptosis still remains unclear. The poly(ADP-ribose) polymerase (PARP) pathway can also activate this mode of programmed necrosis. PARP-1 activation in response to excessive DNA damage results in the massive synthesis of poly(ADP-ribose) polymers (PAR), NAD^+^ depletion and subsequent release of apoptosis inducing factor (AIF) from mitochondria, which translocates to the nucleus where it forms an active DNA-degrading complex (caspase-independent pathway). The PARP pathway has been considered as an integral part of TNF-induced necroptosis; however, it has been recently described that both pathways represent distinct and independent routes to programmed necrosis [22].

The mechanisms responsible for photoreceptor cell death in RP are still unclear. However, increasing evidence suggests that inflammation [11,12,23,24] and especially TNFα could contribute to the pathogenesis of RP. Therefore, inhibition of TNFα and downstream cellular signaling mechanisms, following interaction of TNFα with its receptors, could be a possible target in the treatment of retinal neurodegenerative disorders such as RP.

In the current study we found that IL-6 and TNFα were increased in the aqueous humor of RP patients. We also observed that pharmacological inhibition of TNFα with Infliximab, a specific monoclonal antibody against TNFα, prevented retinal degeneration in cultures of porcine retina exposed to Zaprinast. This model reproduces some events of the degeneration found in murine models of RP caused by non-functional PDE6 [25]. We also observed in our model a reduction of c*aspase-3* activation, GFAP reactivity and partially oxidative stress, caused by Infliximab treatment. These results suggest that inflammation, especially TNFα upregulation, is playing an important role in retinal degeneration and, importantly, that strategies that promote its blockade could be promising therapies.

## Methods

### Participants in the study

Human samples were obtained, informed consent from all subjects previously having been given. The procedure was in accordance with the tenets of the Declaration of Helsinki and was approved by the IRB of La Fe University Hospital (Valencia, Spain). Thirty adult patients with typical forms of RP characterized by an elevated final dark-adaptation threshold, retinal arteriolar narrowing, and a reduced and delayed electroretinogram were enrolled in the study. Thirteen Caucasian patients suffering from cataracts without any other ocular disease served as controls. Further details of the patients enrolled in the study are shown in Table 1.

**Table 1:** **Description of the participants included in the study**

	**Control**	**RP**
Number of subjects	13	30
Males	7	21
Females	6	9
Age (years)	60 ± 3	48 ± 2

Patients diagnosed of RP were recruited from *Retina Comunidad Valenciana - Asociación Afectados por Retinosis Pigmentaria* and also from the department of Ophthalmology of La Fe University Hospital (Valencia, Spain). Healthy controls were recruited from La Fe University Hospital (Valencia, Spain).

### Ophthalmic examination

The best-corrected visual acuity (BCVA) and automated visual field (VF) were measured in RP patients as previously described [8]. Individual data for each patient is shown in Additional file 1: Table S1. Macular edema secondary to RP was only present in one patient.

### Aqueous humor extraction

Aqueous humor samples from 30 RP patients and from 13 patients with cataracts without any other ocular disease (controls) were collected as previously described [8]. Undiluted aqueous humor samples were collected from each patient, placed in sterile tubes, and stored immediately at −80°C until use. All specimens were assayed to evaluate cytokine concentration in a double-blind arrangement with respect to their group. For each patient, aqueous humors were collected from the eye with the more severe retinopathy.

### Cytokine levels in aqueous humor

The concentrations of cytokines in aqueous humor were measured using a multiplex enzyme-linked immunosorbent assay (ELISA) system. To measure the concentrations of IL-1β, IL-6, IL-10 and TNFα, the SearchLight Custom Human Cytokine-Inflammation Q-Plex Array (Aushon Biosystems, MA, USA) was used. Array was used according to the manufacturer’s instructions. The signal of the cytokine array was determined by a cooled CCD camera (Fujifilm, Tokyo, Japan) using chemiluminescence. SearchLight CCD Imaging and Analysis System were used to quantify cytokine concentrations. The cytokine levels were expressed as pg/mL.

### Porcine retinal explant cultures

Seventy eyes (both left and right eyes from each animal) from small miniature pigs aged 3 to 7 months were obtained from the local slaughterhouse. Neuroretinal explants were carried out as recently described [25]. Treatments were added the day of the culture and maintained for 24 hours. To inhibit PDE6 and induce retinal degeneration, we used a final concentration of 100 nmol/L Zaprinast [25,26]. Zaprinast (Sigma-Aldrich, Madrid, Spain) was diluted in dimethyl sulfoxide (DMSO) (AppliChem, Darmstadt, Germany). The equivalent amount of DMSO was added to the culture medium of controls. To evaluate the possible neuroprotective effect of TNFα blockade we used Infliximab (2 μg/mL, alone or combined with Zaprinast) as TNFα blocker (Remicade®, Schering-Plough, Madrid, Spain). Infliximab is a chimeric human immunoglobulin G1 with a mouse variable fragment having high TNFα affinity and neutralizing capacity.

### Tissue processing and histology

Retinal explants were fixed in 4% filtered paraformaldehyde (Sigma-Aldrich, Madrid, Spain) in 0.1 M PBS (pH 7.4) and cryoprotected in a saccharose gradient (15-20-30%) (Panreac Química, Barcelona, Spain). Samples were frozen embedded in Tissue-Tek® OCT™ Compound (Sakura Finetek Europe BV, Zoeterwoude, The Netherlands). After this, 10-μm sections were cut with a cryostat (Leica CM1900, Nussloch, Germany) and placed on Super Frost Ultra Plus treated slides (Thermo Scientific, Barcelona, Spain).

### TUNEL assay

To evaluate apoptosis the terminal deoxynucleotidil transferase dUTP nick and labeling (TUNEL) assay was used as previously described [25]. The apoptotic (TUNEL-positive) nuclei per field were counted in at least three fields per retinal explant using NIS-Elements imaging software (NIKON Instruments, Badhoevedorp, The Netherlands). The number of apoptotic nuclei was normalized to the SYTOX Green-labeled cell nuclei. Results are given as percentage of apoptotic nuclei/total nuclei. Data are expressed as mean ± SEM.

### Immunofluorescence of *caspase-3,* GFAP and PAR

Immunofluorescence was carried out on 10-μm cryosections. Sections were post-fixed for 15 minutes at room temperature in 4% filtered paraformaldehyde (Sigma-Aldrich, Madrid, Spain) in 0.1 M PBS (pH 7.4). Sections were incubated for 1 hour in blocking solution containing 5% normal goat serum, 1% BSA and 0.25% Triton X-100. They were then incubated with primary antibody against cleaved *caspase-3* (1:200, Cell Signaling Technology, Barcelona, Spain), glial fibrillary acidic protein (GFAP, 1:400, Sigma-Aldrich, Madrid, Spain) or PAR (1:200, Enzo Life Science, Madrid, Spain) overnight at 4°C in blocking solution. After this samples were incubated for one hour at room temperature with the fluorescence-conjugated secondary antibody Alexa Fluor 647 (Invitrogen, Life Technologies, Madrid, Spain) and observed under a confocal microscope (Leica TCS SP5 Confocal microscope, Leica Microsistemas SLU, Barcelona, Spain) belonging to the Microscopy Unit of the IIS-La Fe (Valencia, Spain). Cells were counted at 40× magnification, and the number of *caspase-3* positive cells was counted manually in 4 fields per retinal explant. The number of cells positive for the cleaved *caspase-3* immunolabeling was normalized to the SYTOX Green-labeled cell nuclei (Molecular Probes, Paisley, UK). Results are given as percentage of *caspase-3* positive cell/total nuclei. Data are expressed as mean ± SEM.

GFAP and PAR positive cells were difficult to count in different retinal layers. For the quantification we used the following formula to calculate the corrected fluorescence (CF) for each cell layer [27]:

$$ \mathrm{C}\mathrm{F}=\mathrm{Integrated}\;\mathrm{density}\;\mathrm{of}\;\mathrm{the}\;\mathrm{selected}\;\mathrm{area}\hbox{-} \left(\mathrm{area}\;\mathrm{of}\;\mathrm{selected}\;\mathrm{area}\times \mathrm{mean}\;\mathrm{fluorescence}\;\mathrm{of}\;\mathrm{background}\right) $$

Data are expressed as mean ± SEM.

For co-localization of cleaved *caspase-3* (combined with Alexa Fluor 647) and PAR (combined with Alexa Fluor 488 (Invitrogen, Life Technologies, Madrid, Spain)) staining was followed by TUNEL staining.

### *caspase-3* activity assay

*caspase-3* activity was measured with a colorimetric tetrapeptide (DEVD-*p*NA) cleavage assay kit following the manufacturer’s instructions (Bio-Vision, Mountain View, CA, USA). Total retinal protein was extracted from retinal explants and measured by the bicinchoninic acid (BCA) protein assay. *caspase-3* activity was expressed as arbitrary units (au)/mg of protein.

### Nitrites and nitrates (NOX) determination

Intracellular nitrites (stable end-product of nitric oxide (NO)) and nitrates (NOX) were measured in retinal explants by spectrophotometric GRIESS reaction using nitrate reductase [28]. The tissue NOX levels were expressed as nmol/mg protein.

### Oxidative stress evaluation

Retinal explants were assayed for total antioxidant capacity (TAC) and thiobarbituric acid reactive substances (TBARS) formation as indicator of malonyldialdehyde (MDA) formation.

Retinal explants were homogenized in 5 mM phosphate buffer pH 7, 0.9% NaCl, 0.1% glucose, centrifuged at 10,000 × *g* for 15 minutes at 4°C, and then the supernatants were used to determine TAC and TBARS. Protein concentrations were measured by the BCA protein assay.

TAC was measured using a commercial kit (Cayman Chemical, Ann Arbor, MI, USA) [29]. The tissue TAC levels were expressed as nmol/mg protein.

MDA levels were detected by a colorimetric method involving thiobarbituric acid (TBA) adduct formation (Cayman Chemical, Ann Arbor, MI, USA). Tissue TBARS levels were expressed as nmol/mg protein.

Values for *caspase-3* activity, NOX and oxidative markers are given as the mean ± SEM of at least eight different cultures. For each experiment samples were measured in duplicate.

### Statistical analyses

All statistical analyses were done using R software (version 2.15.3) (Foundation for Statistical Computing, Vienna, Austria). Multivariate analysis of covariance (MANCOVA) and multiple linear regression models were used to analyze human data. For parametric data, ANOVA followed by Newman-Keuls *post hoc* test was used. For non-parametric data, Kruskal-Wallis test followed by Dunn’s Multiple Comparison test was used. Significance levels were set at α =0.05.

## Results

### Increased levels of TNFα and IL-6 in aqueous humor of RP patients

We performed a multiplex ELISA to determine the concentration of TNFα, IL-6, IL-1β and IL-10 in aqueous humor of RP patients. IL-1β and IL-10 were below detectable levels. Descriptive statistics of the results of the measurements of IL-6 and TNFα are shown in Table 2. We performed a MANCOVA with the results of TNFα and IL-6 as dependent variables while disease, age and gender were taken as predictive variables.

**Table 2:** **Protein levels of cytokines in aqueous humor from retinitis pigmentosa (RP) patients and healthy controls**

	**Control**	**RP**
TNF-α (pg/mL)	1.1 ± 0.2	1.7 ± 0.2
95% CI	(0.8 to 1.4)	(1.4 to 2.0)
Detectable samples	13/13	28/30
IL-6 (pg/mL)	10.8 ± 3.4	23.5 ± 3.8
95% CI	(3.2- to 18.5)	(15.8 to 31.3)
Detectable samples	13/13	30/30

Note: values are expressed as mean ± SEM; CI: confidence interval.

**Figure 1:**
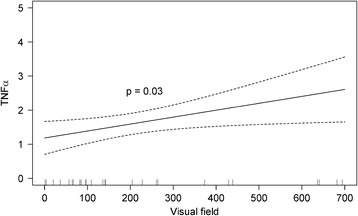
**Relation between visual field and TNFα concentration in aqueous humor of retinitis pigmentosa (RP) patients.** Statistical analysis revealed a positive relation between visual field and TNFα values controlling the other predictive variables (sex, age and acuity). Ninety-five percent confidence intervals are defined by dotted lines.

This analysis revealed that RP significantly increased inflammatory mediators IL-6 and TNFα in aqueous humor (*P* = 0.03) (See Additional file 2: Table S2). We found no statistical evidence for gender or age effects. Further analysis of each of the response variables indicated that IL-6 is increased in RP patients (*P* = 0.018). TNFα showed a tendency to increase in RP patients (*P* = 0.09). We assessed the possible association between inflammatory status (measured as TNFα and IL-6 levels) and stage of the disease (measured as VF and BCVA values) using a MANOVA with VF, BCVA, sex and age as predictors and TNFα and IL-6 levels as response variables. Our results showed no evidence of association between VF and BCVA and inflammatory status (*P* = 0.09 for VF and *P* = 0.94 for visual acuity). Additionally, we also analyzed separately the associations among these predictor variables and each of the two cytokine using linear models. In these analyses we found a statistically significant association between higher VF values and higher levels of TNFα (*P* = 0.03) (Figure 1).

### Infliximab prevents Zaprinast-induced cell death in cultured porcine retina

We previously described that PDE6 inhibition by Zaprinast triggered retinal degeneration and induced oxidative stress and inflammatory mediators such as TNFα and IL-6 in cultured porcine retina after 24 hours. In particular, TNFα and IL-6 content increased to twice control content [25].

We tested whether incubation with 2 μg/mL Infliximab, a TNFα blocker, for 24 hours prevented Zaprinast-induced retinal degeneration. Firstly, we checked the effect of Infliximab on the TNFα signaling cascade. As we could not measure TNFα, because Infliximab interferes with the ELISA assay as previously described [30], we evaluated its receptor TNF-R1 whose activation is involved in multiple apoptotic pathways. TNF-R1 relative expression increased up to 1.36 ± 0.08 arbitrary units (ANOVA Newman-Keuls post-test, *P* <0.0001) in Zaprinast-treated explants compared to control explants (1.00 ± 0.08 arbitrary units). However, Infliximab normalized Zaprinast-induced overexpression of TNF-R1 (0.90 ± 0.08 arbitrary units, ANOVA Newman-Keuls post-test, *P* <0.0001). No significant changes were found in explants treated only with Infliximab (0.91 ± 0.06 arbitrary units).

**Figure 2:**
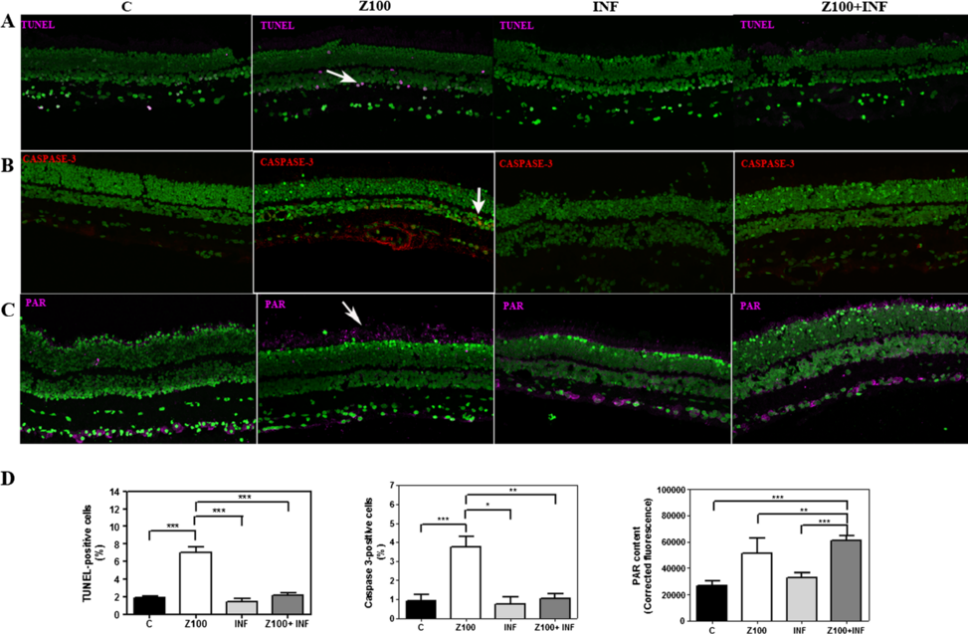
**Infliximab prevents Zaprinast-induced cell death in cultured porcine retina.** Retinal explants were incubated with dimethyl sulfoxide (DMSO), Zaprinast and Infliximab alone or combined with Zaprinast as described in Methods. Confocal laser scanning micrographs of retinal sections showing TUNEL-stained sections visualizing apoptotic photoreceptors (pink) **(A)**, cleaved *caspase-3* positive cells (red) **(B)** and PAR accumulation (pink) **(C)** in SYTOX Green-counterstained retinal sections. Scale bar: 50 μm. **(D)** Bar graphs showing the quantification of TUNEL, cleaved *caspase-3* and PAR accumulation. Values are the mean ± SEM of seven different cultures. Values that are significantly different are indicated by asterisks *P <0.05; **P <0.01; ***P <0.001 (Kruskal-Wallis, Dunn’s post-test). C: control; Z100: 100 μM Zaprinast; INF: 2 μg/mL Infliximab; Z100 + INF: 100 μM Zaprinast with 2 μg/mL Infliximab. TUNEL, terminal deoxynucleotidil transferase dUTP nick and labeling.

As shown in Figure 2, Infliximab significantly reduced the number of TUNEL-positive cells in Zaprinast-treated explants from 7.0 ± 0.7% to 2.2 ± 0.3% (Kruskal-Wallis, Dunn’s post-test, *P* <0.001) (Figure 2A). As shown in Table 3, this reduction occurred mainly in the outer nuclear layer (ONL).

**Table 3:** **Effect of Infliximab treatment on cell death markers in Zaprinast-treated retinal explants**

	**TUNEL-positive cells (%)**				***caspase-3*** **positive cells (%)**				**PAR content (CF)**			
**Layer**	**C**	**Z100**	**INF**	**Z100 + INF**	**C**	**Z100**	**INF**	**Z100 + INF**	**C**	**Z100**	**INF**	**Z100 + INF**
ONL	0.3 ± 0.1	3.0 ± 1.1^a^	0.4 ± 0.2^b^	0.2 ± 0.1^c^	0.07 ± 0.04	0.2 ± 0.1	0.01 ± 0.01^b^	0.04 ± 0.03^c^	7,647 ± 676	22,925 ± 5111^a^	15,982 ± 2,019	24,970 ± 1,807^d,e^
INL	1.1 ± 0.2	2.5 ± 0.4^a^	0.8 ± 0.3^b^	1.1 ± 0.3	0.2 ± 0.1	2.1 ± 0.3^a^	0.4 ± 0.3^b^	0.4 ± 0.1^c^	7,019 ± 1,163	9,348 ± 2,288	11,156 ± 1,879	21,511 ± 2,251^c,d,e^
GCL	0.7 ± 0.1	2.0 ± 0.4^a^	0.6 ± 0.3^b^	1.0 ± 0.3	0.6 ± 0.3	1.3 ± 0.2^a^	0.3 ± 0.1	0.6 ± 0.2^c^	9,891 ± 2,011	10,019 ± 2,212	10,204 ± 1,496	17,134 ± 2,274^c,e^

Note: Kruskal-Wallis test and Dunn’s Multiple Comparisons were used. Values different from control are shown by ^a^(*P* <0.05). Superscripts represent statistical differences (*P* <0.05) between ^b^Z100 and INF; ^c^Z100 and Z100 + INF; ^d^INF and Z100 + INF; ^e^C and Z100 + INF respectively. ONL: outer nuclear layer; INL: inner nuclear layer; GCL: ganglion nuclear layer; PAR, poly(ADP-ribose) polymers; C: control; Z100: 100 μM Zaprinast; INF: 2 μg/mL Infliximab; Z100 + INF: 100 μM Zaprinast with 2 μg/mL Infliximab; CF: corrected fluorescence.

As mentioned above, TNFα can trigger programmed cell death by activating the extrinsic and intrinsic apoptotic pathways that converges on the execution pathway, which is initiated by the cleavage of *caspase-3* [31]. The activity of *caspase-3* in Zaprinast-treated explants was 2.3 ± 0.2 au/mg protein (ANOVA Newman-Keuls post-test, *P* <0.01) and 1.3 ± 0.2 au/mg protein in control explants. Infliximab almost normalized *caspase-3* activity (1.7 ± 0.2 au/mg protein) compared to Zaprinast-treated explants (ANOVA Newman-Keuls post-test, *P* <0.05) and the percentage of cleaved *caspase-3* positive cells (1.1 ± 0.3%) compared to Zaprinast-treated explants (3.8 ± 0.6%, Kruskal-Wallis, Dunn’s post-test, *P* <0.01). Moreover, immunostaining of cleaved *caspase-3* revealed that Infliximab treatment reduced the percentage of *caspase-3* positive cells at all cell layers (outer, inner and ganglion layer (ONL, INL and GCL)) (Kruskal-Wallis, Dunn’s post-test, *P* <0.05) (Table 3 and Figure 2B).

We have previously observed an over activation of poly(ADP-ribose) polymerase (PARP) in our model of porcine retinal degeneration [25]. Moreover, other authors have described similar results in other animal models of retinal degeneration [32,33]. Therefore, we investigated whether TNFα mediated cell death via the PARP pathway. Accumulation of poly(ADP-ribose) polymers (PAR) was used to analyze indirectly PARP activity indirectly. Immunostaining of PAR revealed a significant accumulation of these polymers in ONL and outer segments (OS) in Zaprinast-treated explants (Kruskal-Wallis, Dunn’s post-test, *P* <0.05) that were not prevented by Infliximab treatment (Table 3 and Figure 2C). Infliximab treatment increased PAR accumulation at all cell layers of Zaprinast-treated explants. Thus, the inhibition of TNFα by Infliximab is not causally linked to PARP activation, and therefore does not prevent the secondary PAR accumulation.

**Figure 3:**
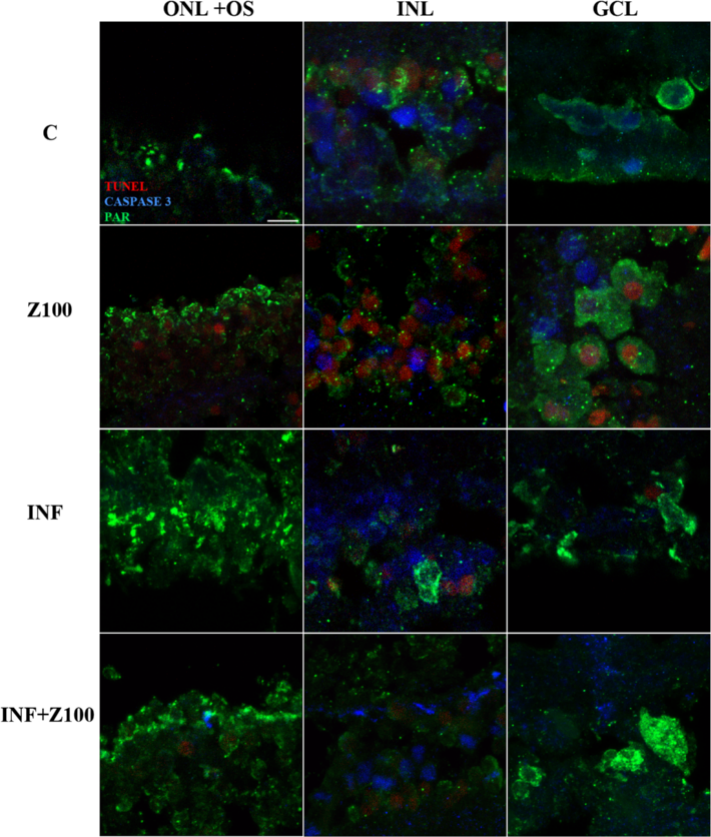
**Co-localization of** ***caspase-3*** **, PAR and TUNEL at different nuclear layers in culture of porcine retina.** Triple-imnunofluorescence labeling of retinal explants treated with dimethyl sulfoxide (DMSO), Zaprinast and Infliximab alone or combined with Zaprinast was carried out as described in Methods. Confocal laser scanning micrographs of retinal sections showing immunolocalization of TUNEL (red), cleaved *caspase-3* (blue) and PAR (green)-positive cells in the nuclear layers of retina. Scale bar: 10 μm. GCL: ganglion nuclear layer; INL: inner nuclear layer; ONL: outer nuclear layer; OS: outer segments; C: control; Z100: 100 μM Zaprinast; INF: 2 μg/mL Infliximab; Z100 + INF: 100 μM Zaprinast with 2 μg/mL Infliximab. TUNEL, terminal deoxynucleotidil transferase dUTP nick and labeling.

To determine whether cleaved *caspase-3* or PAR accumulation co-localize with TUNEL-positive cells, we performed triple labeling (Figure 3). In Zaprinast-treated explants PAR immunostaining co-localized with TUNEL-positive cells in some cells of ONL, in a few cells of the INL and in several cells of GCL. This co-localization disappeared after Infliximab treatment in ONL and GCL but remained in a subset of cells of the INL. PAR accumulation remained high, and even increased, at all cell layers, although the number of TUNEL-positive cells decreased.

However, *caspase-3* positive cells did not co-localize with TUNEL-positive cells except for a subset of cells in INL in Zaprinast-treated explants. Co-localization of *caspase-3* with TUNEL-positive cells disappeared after Infliximab treatment but increased co-localization of *caspase-3* with PAR in INL.

### Infliximab ameliorates Zaprinast-induced glial activation in cultured porcine retina

Gliosis commonly involves upregulation of the intermediate filament protein, GFAP, in Müller glial cells. We studied whether Zaprinast-induced retinal degeneration was accompanied by altered glial reactivity, and if it was the case, whether the blockade of TNFα could prevent it.

**Figure 4:**
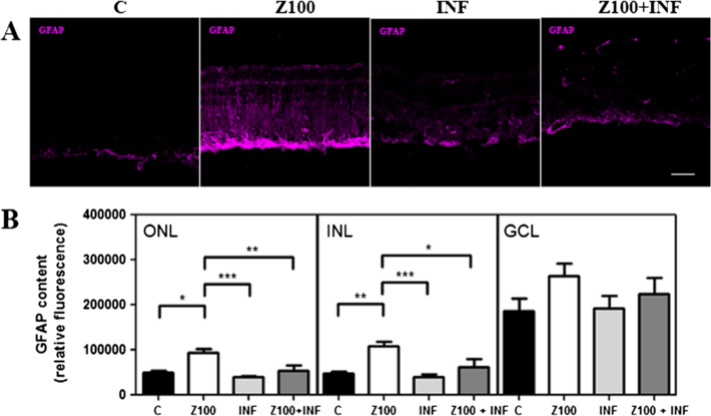
**Infliximab prevents Zaprinast-induced glial fibrillary acidic protein (GFAP) overexpression in cultured porcine retina.** Retinal explants were incubated with dimethyl sulfoxide (DMSO), Zaprinast and Infliximab alone or combined with Zaprinast as described in Methods. **(A)** Confocal laser scanning micrographs of retinal sections showing GFAP content. Scale bar: 50 μm. **(B)** Bar graphs showing the quantification of GFAP content. Values are the mean ±SEM of six different cultures. Values that are significantly different are indicated by asterisks *P <0.05, **P <0.01, ***P <0.001 (Kruskal-Wallis, Dunn’s post-test). C: control; Z100: 100 μM Zaprinast; INF: 2 μg/mL Infliximab; Z100 +INF: 100 μM Zaprinast with 2 μg/mL Infliximab.

In control explants, GFAP were located in the inner half of the retinal Müller cells and their endfeet (GCL layer). However, Zaprinast-treated explants exhibited strong GFAP-positive staining of Müller cells. After PDE6 inhibition, GFAP was massively upregulated throughout the retinal explant. After Infliximab treatment the GFAP-positive labeling was significantly decreased (Figure 4).

### Infliximab partially prevents Zaprinast-induced oxidative stress in cultured porcine retina

cGMP accumulation induces oxidative stress in murine models of retinal degeneration [34] as it does in our model of porcine retina treated with Zaprinast [25]. To explore whether Infliximab also prevented Zaprinast-induced oxidative damage in cultured porcine retina, we measured intracellular nitrite formation (iNOX), as stable NO metabolite, TBARS content as indicator of MDA and total antioxidant capacity (TAC).

**Figure 5:**
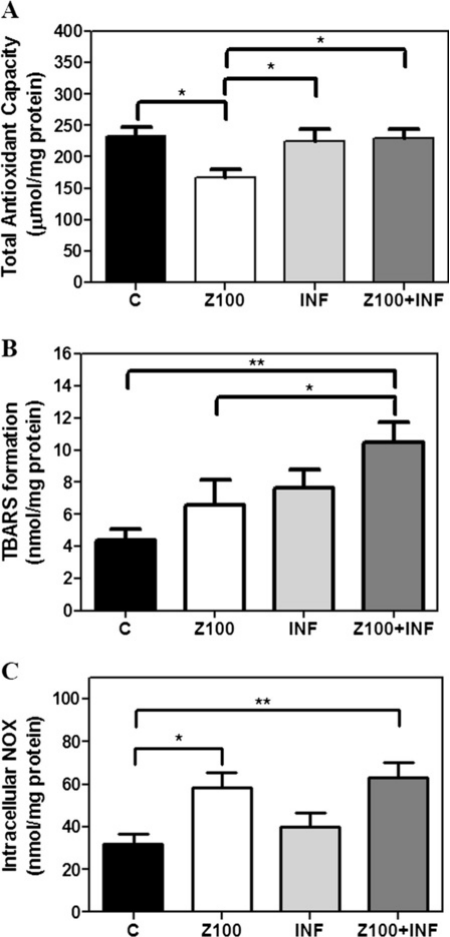
**Infliximab partially prevents Zaprinast-induced oxidative stress in cultures of porcine retina.** Retinal explants were incubated with dimethyl sulfoxide (DMSO), Zaprinast and Infliximab alone or combined with Zaprinast as described in Methods. Effect of Infliximab on the total antioxidant capacity **(A)**, TBARS formation **(B)** and intracellular NOX **(C)**. Each sample was measured in duplicate, and the values are the mean ±SEM of eight cultures. ANOVA Newman-Keuls post-test was used for TAC analysis. Kruskal-Wallis test and Dunn’s post-test was used for TBARS and iNOX analysis. *P <0.05, **P <0.01. C: control; Z100: 100 μM Zaprinast; INF: 2 μg/mL Infliximab; Z100 +INF: 100 μM Zaprinast with 2 μg/mL Infliximab. iNOX, intracellular nitrites and nitrates; TAC, total antioxidant capacity; TBARS, thiobarbituric acid reactive substances.

As shown in Figure 5, Infliximab normalized TAC but did not prevent oxidative stress in Zaprinast-treated explants. Total antioxidant capacity returned to control level (230 ± 15 μmol/mg protein, ANOVA Newman-Keuls post-test, *P* <0.05) (Figure 5A), but TBARS formation (Figure 5B) and intracellular NOX (Figure 5C) remained high after the blockade of TNFα.

## Discussion

Abnormal pathological pathways such as oxidative stress and inflammation, including upregulation of TNFα, have been described in retinal neurodegenerative diseases both affecting the outer retina, such as RP and age-related macular degeneration (AMD), and the inner retina, such as glaucoma and ischemic retinopathy [35-39]. Low-grade inflammation is present in AMD and glaucoma. For instance, in AMD, many mediators of chronic low-grade inflammation such as C-reactive protein, immunoglobulins, and acute phase molecules, the complement-related proteins, autoantibodies, macrophage infiltration and microglial activation have been found [40]. In glaucoma, microglial activation and an inflammatory response involving Toll-like receptors (TLRs), complement molecules and cytokines, such as TNFα and IL-1β, is associated with secondary phase of the disease [41]. Much less is known about the inflammatory response to retinal ischemic-reperfusion (IR) injury. However, pro-inflammatory gene upregulation, accumulation of leukocytes, and microglial activation is found following IR in rodent retinas [42].

In RP, retinal degeneration is caused by various mutations that result in rod death followed by gradual death of cones [43]. Growing evidence suggests that, regardless of the causative mutation, neuroinflammation contributes to photoreceptor degeneration [44,45]. For instance, different animal models of RP (*rds* mice, *rd1* mice, P23 rats, RCS rats) carrying mutations in different genes (*Prph2, PDE6, Rho, Mertk*) show signals of an inflammatory process [23,46-49]. In early stages of retinal degeneration the photoreceptor cells and surrounding cells, such as microglia, respond to unfavourable conditions with the production of cytokines, chemokines, growth factors, and so on, in an attempt to protect neurons and to preserve retinal function. As disease progresses, sustained inflammatory mediators and others such as oxidative stress may exacerbate photoreceptor cell death and RP progression.

Early studies suggested the presence of immune reactivity in RP patients, including the presence of retinal autoantibodies in blood and lymphocytes in vitreous humor. However, these results were variable, maybe due to the inherent genetic heterogeneity of this disease [36]. Afterwards, microglial activation, a common hallmark of both inherited and induced retinal degeneration, was described in RP patients and murine models of RP [12,45,50-53]. It has been shown that microglial activation leads to proliferation, followed by migration to damaged sites and release of cytokines (TNFα, IL-1α, IL-1β) chemokines, neurotrophins, glutamate, NO, superoxide anions and prostaglandins to repair tissue damage. Although these events are triggered to prevent cell damage, sustained high levels of these molecules, especially cytokines, can cause progressive neurodegeneration. In models of RP, microglial activation coincides, or precedes, the peak of photoreceptor cell death and with high levels of TNFα [12,44,45,50,54,55] that seems to be toxic for photoreceptor cells *in vitro* [23]. Besides, microglial inhibition reduces photoreceptor cell death, TNFα content and improves visual function [46].

In our human study we confirmed (1) the presence of high levels of TNFα and IL-6 in aqueous humor in a larger population of RP patients than previously reported [11]; and we observed that (2) RP patients with higher TNFα values show better visual function (visual field). The conflictive positive correlation, between TNFα and better visual function, may be due to the different stage of the disease of the patients. It has been shown that an increase of proinflammatory markers, including TNFα, in mice models of RP occurs just before photoreceptor cell loss [12]. Therefore, it is tempting to speculate that at early onset of RP, when proinflammatory markers are elevated, visual function is better in patients, and after these stages patients lose visual function in parallel with TNFα decrease. In any case, these conflicting, and interesting, results strongly suggest that further studies are needed for clarification.

In the last few years, TNFα has been widely recognized as an attractive therapeutic target for the treatment of retinal diseases. Different types of monoclonal antibodies against TNFα, such as Infliximab, Adalimumab, Certolizumab pegol and Golimumab, or circulating receptor fusion protein, such as Etanercept, have been used to treat glaucoma [56,57], ischemic retinopathy [58] or AMD [59].

The role of TNFα in photoreceptor degeneration and the possible therapeutic use of antibodies against TNFα in the treatment of RP or other retinal degenerations remain quite unexplored. Based on previous studies we decided to evaluate the potential protective effect of the blockade of TNFα in an experimental porcine model of retinal degeneration. In a previous report we demonstrated that this porcine model recapitulated some aspects, especially those related to oxidative stress and inflammation, of the retinal degeneration observed in small animals after PDE inhibition [60,61] and RP patients [8,11]. Sustained elevation of intracellular cGMP in porcine retinal explants triggered different downstream effectors of cell death related to caspase-dependent mechanisms (*caspase-3*) and caspase-independent mechanisms (*calpain-2* and probably PARP activity) [25].

Our current study demonstrated that retinal degeneration accompanied by upregulation of TNFα and IL-6, GFAP and oxidative damage was ameliorated by blocking TNFα with Infliximab. Under our experimental conditions, Infliximab reduced retinal degeneration in all cell layers, mainly in the ONL, by decreasing the number of TUNEL-positive cells, supporting the idea that inflammation plays an important role in the processes of cell death.

We found that Infliximab reduced *caspase-3* activity and the number of cleaved *caspase-3* positive cells across the different cell layers, especially at the INL. Co-localization studies of *caspase-3* and PAR with TUNEL assay suggested that TNFα is promoting cell death through caspase-independent mechanisms in ONL and GCL and caspase-dependent mechanisms in INL.

TNF signaling can lead to cell death to two distinct outcomes, each of which is initiated by different signaling complexes: the apoptosis mode and the necrosis mode. The apoptosis mode includes the extrinsic pathway, mainly mediated by caspases, and the intrinsic or mitochondrial pathway, that rely on the balance between the pro-apoptotic and the anti-apoptotic proteins from the Bcl-2 family. Both pathways converge on the same execution pathway. The execution pathway is initiated by the cleavage of *caspase-3* and results in DNA fragmentation and cell death.

**Figure 6:**
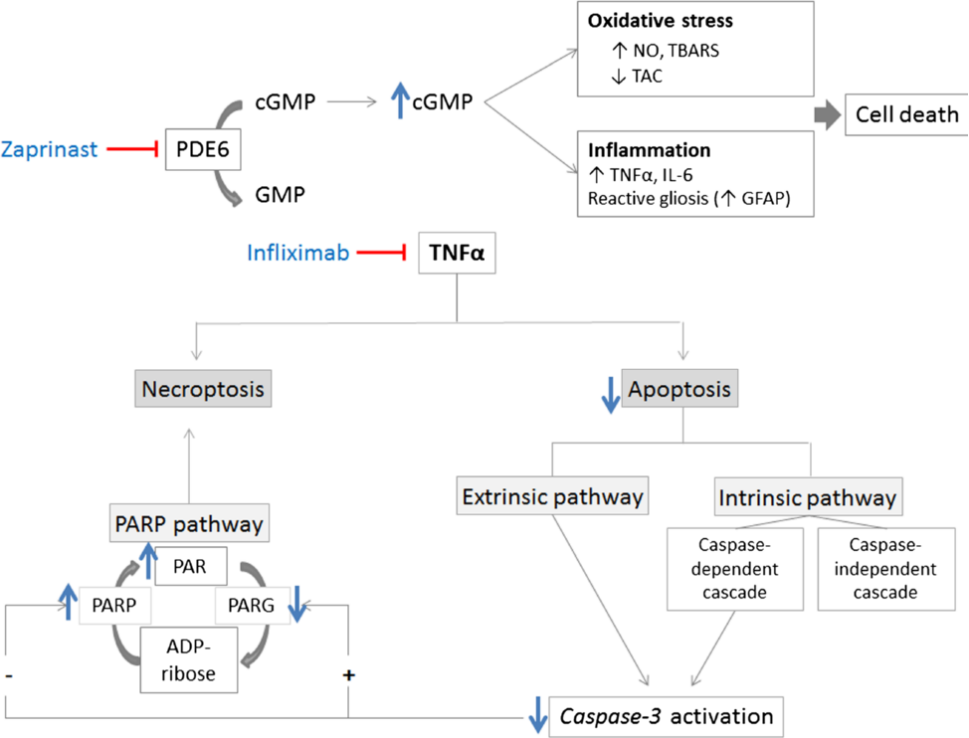
**Diagram showing the possible mechanism of Infliximab in the porcine retinal degeneration model.** PDE6 inhibition induces cGMP accumulation and triggers retinal degeneration. The degeneration is accompanied by upregulation of inflammatory mediators, PARP pathway, reactive gliosis and oxidative stress markers. According to the current study, TNFα may be involved in the retinal degeneration by increasing *caspase-3* activation and reactive gliosis. Infliximab may prevent cell death by inhibiting caspase-dependent pathways that converge in *caspase-3* activation in the INL. Infliximab also may prevent cell death by caspase-independent pathways that remain unclear in the ONL and GCL. Moreover, Infliximab may exacerbate PARP over activation probably through the *caspase-3* inhibition. This over activation could contribute to the future cell death. cGMP: cyclic GMP; GCL, ganglion nuclear layer; GFAP: glial fibrillary acidic protein; INL, inner nuclear layer; NO: nitric oxide; ONL, outer nuclear layer; PAR: poly(ADP-ribose) polymers; PARG: poly(ADP-ribose) glycohydrolase; PARP: poly(ADP)ribose polymerase; PDE6: phosphodiesterase 6; TAC: total antioxidant capacity; TBARS: thiobarbituric acid reactive substances; TNFα: tumor necrosis factor alpha.

We measured indirect activation of PARP through quantification of PAR accumulation. We found an upregulation of PAR due to PDE6 inhibition. However, blockade of TNFα did not prevent PAR accumulation but also increased it. PAR polymers are mainly degraded by poly(ADP-ribose) glycohydrolase (PARG) enzymes, some of them activated by *caspase-3* cleavage [62]. On the other hand, PARP can be inactivated by *caspase-3* cleavage [63]. Therefore, the inhibition of *caspase-3* induced by Infliximab could inhibit PARG activity and prevent PARP inactivation thus exacerbating PAR accumulation at all cell layers of retinal explants. These results support that PARP pathway is independent of TNFα-associated pathways in this experimental model of retinal degeneration (Figure 6).

These results were supported by previous reports in which reactive gliosis (GFAP overexpression) induced by exogenous TNFα was prevented by Adalimumab, other monoclonal anti-TNFα, in a similar model of organotypic culture of porcine neuroretina [64]. Activated Müller cells can release antioxidants, growth factors, and cytokines, including TNFα, contributing to retinal regeneration or to neurodegeneration. Müller cells are activated in models of RP [49,65-68] resulting in overexpression of GFAP, translocation of Müller cell bodies to the outer retina and thickening of their processes [69].

As previously shown, retinal degeneration induced by PDE inhibition was accompanied by oxidative stress in porcine retinas [25]. This is consistent with the idea that oxidative stress is also contributing to the progression of RP in animal models [70-72] and RP patients [8]. In the current study, we demonstrated that Infliximab partially prevented antioxidant defense depletion but not oxidative stress markers. Infliximab normalized the total antioxidant capacity in Zaprinast-treated explants, but it failed to return TBARS and NOX to control levels. In retinas of *rd10* mice antioxidant treatment reduced inflammatory mediators and photoreceptor cell loss [12]. Based on these data, it is tempting to speculate that the low Infliximab effect could be due to oxidative stress preceding upregulation of inflammatory mediators [12,73-75]. The other possibility is that Infliximab affects other oxidative stress markers that we did not measure in this study. Anyway, further studies will be needed to explore this issue in more depth.

In summary, our results corroborate that RP patients have ocular inflammation and that TNFα plays an important role in the retinal degeneration induced by PDE6 inhibition in cultured porcine retinas. The mechanisms of cell death vary in the distinct cell layers. TNFα is involved in retinal degeneration through *caspase-3* activation, caspase-independent mechanisms and reactive gliosis. Our data suggest that other unknown molecules must be contributing to TNFα-mediated cell death in this model. On the other hand, PARP activation is independent of TNFα signaling and it is probably responsible for a future cell death in ONL. The existence of several distinct pathways that trigger programmed cell death implies that an efficient protection requires their simultaneous interruption via combined therapies.

The experimental model of organotypic culture has its own limitations because it involves transection of the optic nerve and mechanical retinal detachment causing retrograde retinal ganglion cell degeneration. To minimize this problem, we have used detached retinas as controls. Moreover, the model cannot recapitulate the whole chronic nature of the degeneration, but we believe that it could be useful for studying some aspects related to the retinal degeneration. In our case, we believe that it may provide a helpful model to design and assay some treatments, such as Infliximab, thus replacing or reducing animal experiments. The use of this model allowed us to evaluate the effect of Infliximab faster and more cheaply than using the available *in vivo* models of RP.

The current model of retinal degeneration allowed us to describe an interesting and, in our opinion, neuroprotective effect of Infliximab that strongly encourages further exploration using other experimental models. Due to the importance of the inflammatory process in the pathogenesis of several retinal degenerative conditions such as RP, AMD, ischemic retinopathy, or glaucoma, targeting inflammation could be a promising therapeutic strategy. In particular, TNFα blockers could be a new therapeutic strategy for the treatment of RP and other retinal degenerative conditions.

## Additional files

Additional file 1: Table S1. Individual data for each patient with retinitis pigmentosa (RP). Additional file 2: Table S2. MANCOVA in aqueous humor from retinitis pigmentosa (RP) patients and healthy controls.

## Abbreviations

AIF: apoptosis inducing factor
AMD: age related macular degeneration
ANCOVA: analysis of variance
au: arbitrary units
BCVA: best-corrected visual acuity
BCA: bicinchoninic acid
BSA: bovine serum albumin
cGMP: cyclic guanosine monophosphate
DMSO: dimethyl sulfoxide
ELISA: enzyme-linked immunosorbent assay
GCL: ganglion nuclear layer
GFAP: glial fibrillary acidic protein
IL: interleukin
INL: inner nuclear layer
iNOX: intracellular nitrite
IR: ischemic-reperfusion
MANCOVA: multivariate analysis of covariance
MDA: malonyldialdehyde
NO: nitric oxide
NOX: intracellular nitrates and nitrites
ONL: outer nuclear layer
PAR: poly(ADP-ribose)
PARP: poly(ADP-ribose) polymerase
PBS: phosphate-buffered saline
PDE6: phosphodiesterase 6
RP: retinitis pigmentosa
TAC: total antioxidant capacity
TBA: thiobarbituric acid
TBARS: thiobarbituric acid reactive substances
TLRs: Toll-like receptors
TNFα: tumor necrosis factor alpha
TUNEL: terminal deoxynucleotidil transferase dUTP nick and labeling
VF: visual field
