# Supplementary material for: Infliximab reduces Zaprinast-induced retinal degeneration in cultures of porcine retina
Source: J Neuroinflammation. 2014 Oct 10;11:172. doi: 10.1186/s12974-014-0172-9 (PMC4200228; doi:10.1186/s12974-014-0172-9)
Supplement: Additional file 1: Table S1. — Individual data for each patient with retinitis pigmentosa (RP). [file 12974_2014_172_MOESM1_ESM.doc]

**Table S1 Individual data for each patient with retinitis pigmentosa (RP)**

| Patient | | Age (year) | Sex | | Acuity of worst eye (logMAR) | Visual Field of worst eye  (dB) | Type of RP | Type of inheritance/Gene |
| --- | --- | --- | --- | --- | --- | --- | --- | --- |
| 1 | 43 | | | M | 0.3 | 4 | NS | Unknown |
| 2 | 45 | | | M | 0.0 | 1 | NS | ARRP |
| 4 | 56 | | | M | 0.6 | --- | NS | Unknown |
| 5 | 57 | | | M | 0.05 | 37 | NS | Unknown |
| 6 | 37 | | | M | 0.7 | 439 | NS | Unknown |
| 7 | 54 | | | F | 0.4 | 261 | NS | Unknown |
| 8 | 35 | | | F | 0.5 | 694 | NS | Unknown |
| 9 | 57 | | | M | 0.2 | 85 | NS | SRP |
| 10 | 39 | | | M | 0.5 | 682 | NS | ADRP |
| 11 | 35 | | | M | 0.4 | 636 | NS | ARRP |
| 12 | 34 | | | F | 0.5 | 640 | NS | ADRP |
| 30 | 47 | | | M | --- | --- | S | AR/*USH2A* |
| 31 | 44 | | | F | 0.1 | 65 | NS | ADRP |
| 33 | 48 | | | F | 0.05 | 109 | S | AR/*USH2A* |
| 35 | 57 | | | M | 1.0 | 228 | NS | Unknown |
| 37 | 51 | | | F | 0.05 | 97 | NS | Unknown |
| 39 | 47 | | | M | 0.7 | 204 | NS | Unknown |
| 40 | 45 | | | F | 0.05 | 141 | S | AR/*USH2A* |
| 41 | 63 | | | M | 0.1 | 95 | NS | Unknown |
| 44 | 48 | | | M | 0.2 | 82 | NS | Unknown |
| 45 | 51 | | | M | 0.05 | 57 | S | AR/*USH2A* |
| 46 | 35 | | | F | 0.05 | 20 | NS | ARRP |
| 48 | 46 | | | M | 0.8 | 264 | S | AR/*USH2A* |
| 50 | 39 | | | M | 0.4 | 97 | NS | Unknown |
| 53 | 53 | | | M | 0.6 | 429 | NS | ADRP |
| 54 | 39 | | | F | 0.8 | 85 | NS | Unknown |
| 55 | 68 | | | M | 0.05 | 67 | NS | Unknown |
| 56 | 58 | | | M | 0.2 | 136 | NS | Unknown |
| 57 | 66 | | | M | 0.7 | 373 | NS | ADRP |
| 59 | 39 | | | M | 1.0 | 143 | NS | Unknown |

Note: RP: retinitis pigmentosa; NS: non-syndromic; S: Syndromic; ARRP, autosomic recessive RP; ADRP, autosomic dominant RP; SRP, sporadic RP; AR: autosomic recessive; *USH2A*: Usher syndrome type 2A.
