# Supplementary material for: Infliximab reduces Zaprinast-induced retinal degeneration in cultures of porcine retina
Source: J Neuroinflammation. 2014 Oct 10;11:172. doi: 10.1186/s12974-014-0172-9 (PMC4200228; doi:10.1186/s12974-014-0172-9)
Supplement: Additional file 2: Table S2. — MANCOVA in aqueous humor from retinitis pigmentosa (RP) patients and healthy controls. [file 12974_2014_172_MOESM2_ESM.doc]

**Table S2 MANCOVA in aqueous humor from retinitis pigmentosa (RP) patients and healthy controls.**

| **Predictive Variables** | **Pillai´s trace** | **P-value** |
| --- | --- | --- |
| **Disease RP** | 0.18 | 0.033* |
| **Age** | 0.14 | 0.081 |
| **Gender** | 0.03 | 0.547 |

**Note**: Pillai´s trace, multivariate test criteria used in the multivariate analysis of covariance (MANCOVA).
